# New feature extraction from phylogenetic profiles improved the performance of pathogen-host interactions

**DOI:** 10.3389/fcimb.2022.931072

**Published:** 2022-08-02

**Authors:** Yang Fang, Yi Yang, Chengcheng Liu

**Affiliations:** ^1^ Key Laboratory of Bio-Resources and Eco-Environment of Ministry of Education, College of Life Sciences, Sichuan University, Chengdu, China; ^2^ Department of Laboratory Medicine, Third Affiliated Hospital of Zhengzhou University, Zhengzhou, China; ^3^ State Key Laboratory of Oral Diseases, Department of Periodontics, National Clinical Research Center for Oral Diseases, West China School & Hospital of Stomatology, Sichuan University, Chengdu, China

**Keywords:** pathogen-host interaction, machine learning, phylogenetic profile, virus, bacteria

## Abstract

**Motivation:**

The understanding of pathogen-host interactions (PHIs) is essential and challenging research because this potentially provides the mechanism of molecular interactions between different organisms. The experimental exploration of PHI is time-consuming and labor-intensive, and computational approaches are playing a crucial role in discovering new unknown PHIs between different organisms. Although it has been proposed that most machine learning (ML)–based methods predict PHI, these methods are all based on the structure-based information extracted from the sequence for prediction. The selection of feature values is critical to improving the performance of predicting PHI using ML.

**Results:**

This work proposed a new method to extract features from phylogenetic profiles as evolutionary information for predicting PHI. The performance of our approach is better than that of structure-based and ML-based PHI prediction methods. The five different extract models proposed by our approach combined with structure-based information significantly improved the performance of PHI, suggesting that combining phylogenetic profile features and structure-based methods could be applied to the exploration of PHI and discover new unknown biological relativity.

**Availability and implementation:**

The KPP method is implemented in the Java language and is available at https://github.com/yangfangs/KPP.

## Introduction

Pathogen-host interactions (PHIs) are crucial for understanding the interactions between different organisms. Most diseases in humans are caused by the virus (Brass et al., 2008; McDermott et al., 2012), and knowing the mechanisms of human PHI is important for developing effective therapeutics. In the study of plants, pathogen infections reduce crop yields (Bernardes-de-Assis et al., 2009; Savary et al., 2012). Understanding the PHI in plants is essential for the defense against plant diseases. The early analyses were built on yeast by the yeast two-hybrid approach (Uetz et al., 2000; Ito et al., 2001). This method provided an experimental way to explore protein-protein interactions in yeast cells. However, exploring PHI based on experimental methods is time-consuming and expensive, and computational methods play an important role in complementing the experimental methods. Over the past decade, various methods have been proposed for deciphering PHI. These include structure-based methods (Shen et al., 2007; Guo et al., 2008; Zhou et al., 2012), homology-based methods (Krishnadev and Srinivasan, 2011; Wuchty, 2011), domain-motif approaches (Dyer et al., 2007; Evans et al., 2009), and machine learning–based (ML-based) methods (Qi et al., 2010; Dyer et al., 2011).

With an increasing number of experimental PHI data being published, many databases have been developed to collect and store these PHI data (Ako-Adjei et al., 2015; Calderone et al., 2015; Guirimand et al., 2015; Urban et al., 2017). Because a large number of experimental PHIs are available, it is possible to use experimental data to drive supervised ML-based methods to predict PHI. For example, Yang et al. used four structure-based feature methods and one network-based feature vector trained by the random forest (RF) method to increase the prediction accuracy of plant PHIs (Yang et al., 2019). Abbsali et al. encoded human and hepatitis C virus proteins as feature vectors by six different descriptors trained by four different ML-based methods that achieved high accuracy and specificity (Emamjomeh et al., 2014). Xianyi et al. extracted five structure-based features with the ML method to predict human and bacterial interactions (Lian et al., 2019). Therefore, extracting protein information features from different methods can significantly improve the prediction results of PHI. Although features can be extracted from various information or evidence for predicting PHI by ML-based methods, most ML-based methods generate features from protein sequence information.

For the first time, the phylogenetic profile was used to predict gene function based on homologies of a reference genome across organisms (Pellegrini et al., 1999). The phylogenetic profile plays a critical role in exploring gene functions (Eisen and Wu, 2002; Jiang, 2008; Li et al., 2014). In addition, the phylogenetic profile has been widely explored in the protein-protein interactions (Pellegrini et al., 1999; Date and Marcotte, 2003; Wu et al., 2003). We first combined the phylogenetic profile and the ML method to explore the PHI. The features extracted from the phylogeny can better reflect the homology relationship in the evolution of the various organisms.

We provide a new method named KPP (kmer phylogenetic profile) that extracts features from the phylogenetic profile for the ML-based method–predicted plant PHI. Our methods construct phylogenetic profiles by contig information and extend phylogenetic profiles by five various models [based on properties of amino acids (AAs)]. We concatenate the phylogenetic feature, and structure-based features significantly improved the prediction results suggesting that the descriptor features extracted from the phylogenetic profile are very important information for predicting plant PHI. In addition, the test results showed that the KPP method can also be applied to the PHI prediction of human bacteria and human viruses. The KPP method is implemented in the Java language (which supports Linux, Windows, and Mac OS platforms) and is freely accessible from the Github repository (https://github.com/yangfangs/KPP).

## Results

### Extracting phylogenetic profile features for predicting plant PHI

**Figure 1:**
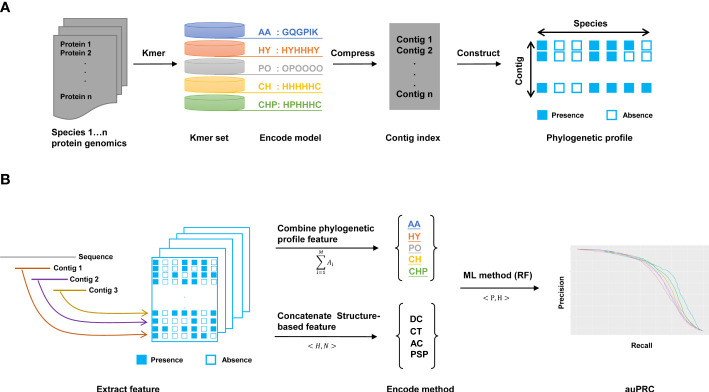
The workflow of this work. **(A)** Construction of the kmer phylogenetic profile. Each protein sequence was cut to the kmer set and compressed to the contig index to construct the phylogenetic profile. There are AA, HY, PO, CH, and CHP models for building kmer phylogenetic profiles. **(B)** Extracting features and predicting by the ML method. There are two ways to merge features. One is the “combine method” for merging five models to extract features from phylogenetic profiles. Another is the “concatenate method” for the structure-based method. In this work, we use the RF method to predict plant PHI.

Here, we design a method named KPP that extracts features from phylogenetic profile to predict the interaction of plant pathogens and hosts (**Figure 1**). First, we build the contig index by kmer. We split each AA sequence into a kmer set and searched the consensus region of this kmer as contig index (Gregory, 2001). Using contigs as an index can effectively compress data compared to kmer while reducing the number of retrievals when extracting features and improving computational efficiency (**Supplementary Figure 1**). Second, we constructed the phylogenetic profile by the contig index; in this step, the rows and columns of the phylogenetic profile are represented by contigs and species, respectively (**Figure 1A**). Moreover, there were five different models used to build the phylogenetic profile. The AA profile is constructed by amino acids. The HY profile is constructed based on the hydrophilic and hydrophobic properties of AAs. The PO profile is driven by the polar properties of AAs, and the CH profile is built by the charged properties of AAs. The HY&PO&CH(CHP) profile concatenates three different properties of AAs to build a phylogenetic profile. The classification of various models based on the 20 common AAs has their specific chemical characteristics and their different roles in protein structure and function (Scheiner et al., 2002) are summarized in **Supplementary Table 1**. As shown in **Figure 1B**, we extracted features from binary phylogenetic profiles that combine or concatenate various method features to predict plant PHI. We trained this feature by the ML-based method; here, we use RF as a classifier to predict the interaction of PHI. Additionally, the area under the precision-recall curve (auPRC) is used as an indicator to evaluate the quality of the model.

### The phylogenetic profile feature is significant for ML

**Figure 2:**
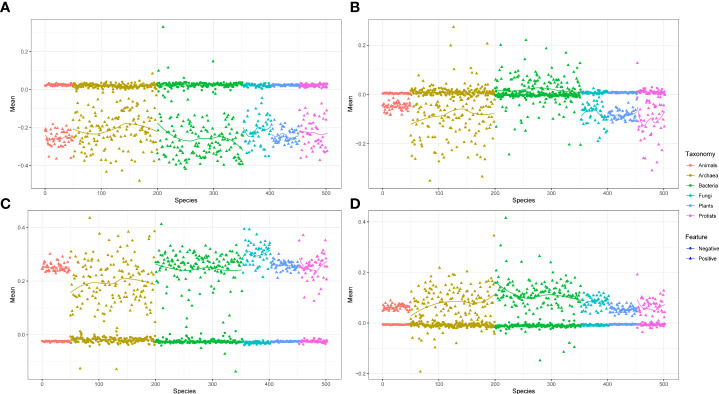
The distribution of positive and negative train feature data based on phylogenetic profile. **(A)** The feature data distribution of Gor-Ara. **(B)** The feature data distribution of Psy-Ara. **(C)** The feature data distribution of Hpa-Ara. **(D)** All-Ara feature data. All of these features were extracted from the AA model with 503 species and the kmers setting with 6. The red and blue dots represent negative and positive data, respectively.

The phylogenetic profile provided significant data features for the ML training. In this study, we chose three different pathogens *Golovinomyces orontii* (Gor), *Hyaloperonospora arabidopsidis* (Hpa), and *Pseudomonas syringae* (Psy), and also *Arabidopsis thaliana* (Ara) as the host plant (Mukhtar et al., 2011; Wessling et al., 2014). These three pathogen species and one plant species comprised the Gor-Ara, Hpa-Ara, and Psy-Ara test datasets, respectively. Gor and Hpa are eukaryotic pathogens that contain 122 and 104 positive pairs, respectively. Psy is a prokaryotic pathogen that contains 233 positive pairs. The negative pairs are 10 times as large as the positive pairs generated from random pairs in each species (Yang et al., 2019). We used the KPP algorithm to generate the kmer set to construct the contig index and phylogenetic profile. We extracted the feature from the phylogenetic profile and normalized this feature by the z-score method. The mean of these positive and negative feature data is presented in **Figure 2** (taxonomy by phylum). As shown in **Figure 2**, all the test data show that the mean value of the feature of the negative data is stable at 0, and the positive data will fluctuate up and down the negative data and have significant differences (Mann–Whitney two-tailed test p-value< 10^−8^). This difference is most obvious in the interaction between eukaryotic pathogens and Ara. (**Figures 2A, C**). The results suggest that the extract profile from the phylogenetic profile can be used to distinguish the positive and negative pairs of each pathogen to Ara. A strong predicted true pair sample by phylogenetic profile feature was observed (**Supplementary Figure 2**). The predicted probability shows that negative test samples appear in the probability interval of 0 to 0.5. In the probability interval greater than 0.7, only the predicted results of the positive test samples are available. This indicates that the feature values extracted from the phylogenetic profile can better separate the positive and negative test results and have higher precision.

### The performance of the KPP algorithm

#### The performance of the five models

**Figure 3:**
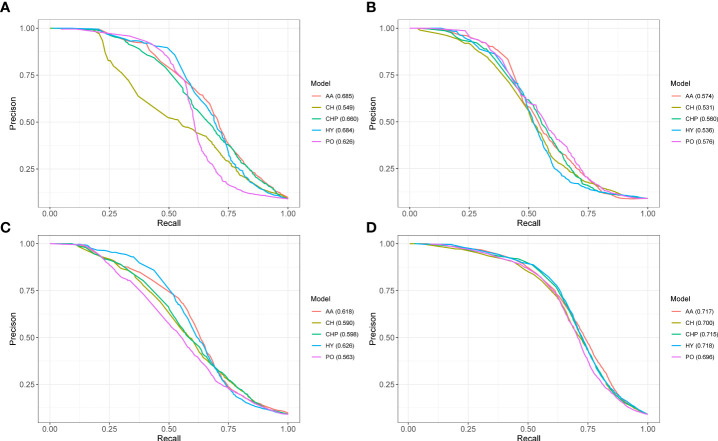
The performance of phylogenetic profile features predicted pathogen-host interactions. PR curves show the performance of five different models on the 10-fold cross-validation test. Panels **(A–D)** represent the results from the Ara-Psy, Ara- Hpa, Ara-Gor, and All-Ara training samples, respectively.

Here, we test five different models by 10-fold cross-validation and the PR curves illustrated in **Figure 3**. From PR curves, we can see that the auPRC of all predicted models greater than 0.5 indicates that the feature extracted from the phylogenetic profile can distinguish positive and negative data well. The performance of the three plant PHI test datasets showed that Psy-Ara (aucPRC = 0.685 for AA model) performed better than the Hpa-Ara (aucPRC = 0.574 for AA model) and Gor-Ara (aucPRC = 0.618 for AA model) species in the test. What is interesting about the test sample in **Figure 3D** is that, as the test sample set increases (All-Ara), the performance results of the five models have improved. The auPRC values all exceeded 0.7 except for the PO model (**Figure 3D**). These results suggest that the phylogenetic profile features are a powerful indicator that can distinguish whether there is an interaction between pathogens and hosts in plant PHI.

#### Parameter optimization for performance

We used contigs and species to construct phylogenetic profiles and extract features for ML to predict plant PHI. The length of the kmer and the selection of the species number are critical to the performance of the prediction results. We use different numbers of species to construct a phylogenetic profile (test with AA model, k = 6 and randomly chose the species with 72, 503, and 1,000) (**Supplementary Table 2**). The results show that the performance of the predicted results increased as the number of species increased. Too many species chosen will reduce the speed of contigs searches, so based on the balance of calculation time and accuracy, we chose 503 species as the optimal species selection for constructing phylogenetic profiles (**Supplementary Figure 3** and **Supplementary Table 3**). Due to the different properties of AAs, we encode AA characters into four different models, which will lead to the optimal length of kmer for each model being various. We tested kmer length against different models to select the optimal kmer value with 503 species (**Supplementary Table 4**). The result clearly shows that for the AA, HY, PO, CH, and CHP models, the optimal kmer values are 6, 22, 27, 19, 15, respectively. The following tests on the algorithm are based on these optimal parameters.

### The phylogenetic profile feature significantly improved the performance of ML prediction

**Figure 4:**
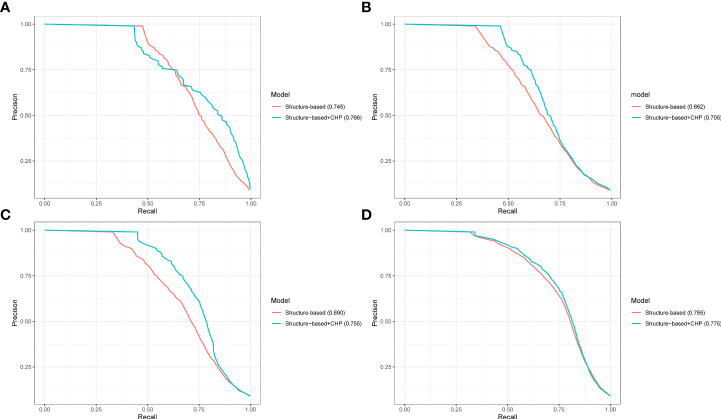
The performance of merging different features predicted pathogen-host interactions. PR curves showing the performance based only on the structure-based and structure-based + CHP models on the 10-fold cross-validation test. Panels **(A–D)** represent the results from the Ara-Psy, Ara- Hpa, Ara-Gor, and All-Ara training samples, respectively.

We concatenate novel phylogenetic profile features (CHP model) with sequence features to improve the performance of prediction in the plant PHI. To compare the influence of phylogenetic profile features on the performance, we compared the structure-based + CHP with the structure-based descriptions (CT, AC, DC, and PSP descriptions in the Methods section) based on the RF algorithm. As shown in **Figure 4**, the aucPRC values of the structure-based + CHP method in the 10-fold cross-validation test were 0.766, 0.705, 0.755, and 0.775 for the Gor-Ara, Psy-Ara, Hpa-Ara, and All-Ara test data, respectively, whereas the corresponding values of the structure-based method were 0.745, 0.662, 0.690, and 0.765, respectively. In addition, the performance of the other models (AA, CH, PO, and HY) + the structure-based model is shown in **Supplementary Table 5**. The results show that by concatenating the feature extracted from the phylogenetic profile with the structure-based feature to predict plant PHI, five different models can improve the performance of the prediction results. It also shows that the phylogenetic profile is a significant feature for the prediction of plant PHI based on the ML method. In general, the structure-based + CHP feature was reported significantly more often than the structure-based descriptor only. The results of cross-validation clearly show that phylogenetic profile features can substantially improve the predicted performance of plant PHI. The traditional method uses the concatenate method to connect different features to improve the dimensionality of the training feature value and improve the accuracy (Emamjomeh et al., 2014; Yang et al., 2019; Yang et al., 2020). Strikingly, because the features extracted from the phylogenetic profile by five models have the same dimensions (503), we proposed a “combine” method to merge feature values for ML. The merged value dimension has not increased, and the length of the feature is still 503, which greatly reduces the calculation pressure and improves the prediction speed. At the same time, the performance of our “combine” method (combine AA, HY, PO, CH, and CHP features) is better than that of the traditional concatenate method (**Supplementary Figure 4**).

Here, we use the RF method as the main ML algorithm to predict plant PHI because it performed better than the other ML methods. We also compared corresponding results with different ML algorithms, including support vector classifier (SVC), gradient boosting classifier (GBC), K-neighbors classifier (KNC), AdaBoost classifier (ADB), and Naive Bayes (NB) (**Supplementary Figure 5**). These algorithms were implemented by the Python-based library Scikit-learn (Pedregosa et al., 2011). We found that RF (auPRC = 0.715) obtained the best performance in the All-Ara test dataset, followed by ADB (auPRC = 0.609) and GBC (auPRC = 0.560). However, the SVC (auPRC = 0.450), KNC (auPRC = 0.368), and NB (auPRC = 0.167) methods obtained the worst performance and were not applicable to plant PHI prediction (**Supplementary Figure 5D**). There was a similar performance ranking in the other three test datasets (**Supplementary Figures 5A–C**). This result suggested that the RF method was the best appropriate ML algorithm for predicting plant PHI, and we used this method to train phylogenetic profile features for predicting plant PHI.

### The performance of the KPP feature in human PHI

**Figure 5:**
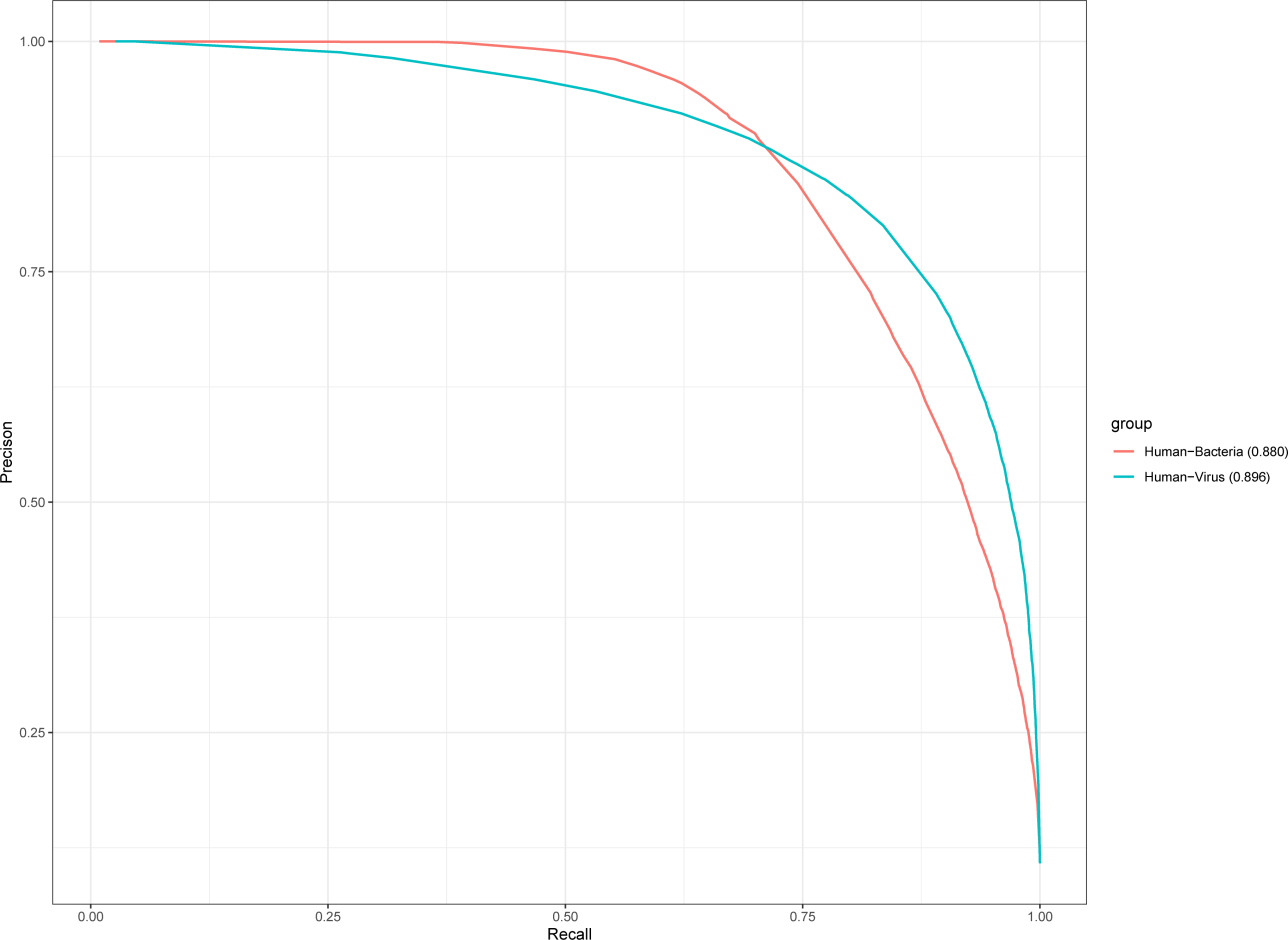
The performance of the KPP method predicted human bacteria and human viruses. Ten-fold cross-validation constructs auPRC for human bacteria and human viruses.

We validate the performance of the KPP method in human PHI by human bacteria (13,413 positive pairs) and human virus (14,789 positive pairs). The human-bacteria PHI and human-virus–positive were collected from HPIDB 3.0 database (Ammari et al., 2016). The human-virus PHI contains six virus species (*influenza A virus*, *human papillomavirus type 16*, *measles virus*, *Zika virus*, *HIV-1 M:B_HXB2R*, and *human herpesvirus*). In this test dataset, *influenza A virus* was the most positive pair among these six species including 6,070 positive pairs. The species with the least number of positive pairs was the *measles virus*, which contained a total of 906 positive pairs (**Supplementary Table 6**). The human-bacteria PHI contains five bacterial species (*Yersinia pestis*, *Bacillus anthracis*, *Francisella tularensis*, *Saccharomyces cerevisiae*, and *Streptococcus pyogenes*). Because there is no database of PHI for the oral cavity, we collected experimental human–oral bacteria PHI (Rosa et al., 2020). We extracted 485 bacteria that inhabited in the human oral cavity from the eHOMD database (Chen et al., 2010). We checked these oral bacteria to human interactions from the DIOGRID database (Stark et al., 2006), IntAct database (Kerrien et al., 2012), and HPIDB3.0 database (Ammari et al., 2016). However, we only identified 13 positive pairs in *Streptococcus pyogenes* bacteria as human oral bacteria (**Supplementary Table 6**). We test the performance of KPP features in human bacteria and various by 10-fold cross-validation and the auPRC shown in **Figure 5**. As shown in **Figure 5**, the auPRC of human bacteria is 0.880, and the auPRC of human viruses is 0.896. Strikingly, the performance of the KPP method in animal PHI tests is better than that in plant PHI tests. This result suggested that the KPP feature improves not only the performance of plant PHI but also that of human-bacteria and human-virus PHI.

### Prediction of PHI between humans with viruses and bacteria by the KPP method

We used the KPP method extract feature to predict human-virus and human-bacteria PHIs with the RF method. As shown in **Table 1**, the three viruses (*human herpesvirus 4 strain B95-8*, *Zika virus*, and *influenza A virus*) and bacteria (*Bacillus anthracis*, *Yersinia pestis*, and *Glossosomatidae)* reported significantly predicted results. However, *HIV-1 M:B_HXB2R*, *measles virus strain Schwarz*, *and Saccharomyces cerevisiae* S288C did not obtain significant prediction results with the 0.6 predicted cutoff. *Human herpesvirus 4 strain B95-8*, *Zika virus*, and *influenza A virus* predicted significant pairs of PHIs of 19, 2, and 19, respectively, with a cutoff of 0.6 (**Supplementary Table 7**). The predicted pairs of PHIs for *Bacillus anthracis*, *Yersinia pestis*, and *Glossosomatidae* were 24, 295, and 144, respectively (**Supplementary Table 8**). Because human-bacteria PHI obtained a lower AUC performance in the training dataset, we chose a higher threshold value at the time of prediction.

**Table 1:** Prediction of the PHIs between humans with viruses and bacteria by the KPP method.

Species	Taxonomy ID	Train AUC	Predicted pairs	Cutoff
*Human herpesvirus 4 strain B95-8*	10377	0.874	19	0.600
*Zika virus*	64320	0.770	2	0.600
*Influenza A virus*	381518	0.842	19	0.600
*HIV-1 M:B_HXB2R*	11706	0.932	NA	0.600
*Measles virus strain Schwarz*	132487	0.970	NA	0.600
*Bacillus anthracis*	1392	0.730	24	0.700
*Yersinia pestis*	632	0.700	295	0.850
*Glossosomatidae*	177416	0.653	144	0.700
*Saccharomyces cerevisiae* S288C	559292	0.994	NA	0.600

## Discussion

In this work, we developed a KPP method to extract phylogenetic profile features for predicting plant PHI. The KPP method provides five models to construct a phylogenetic profile based on the properties of AAs. Because the feature dimensions extracted from five various phylogenetic profiles are the same, we first proposed a method of longitudinally merging features to keep the feature dimensions unchanged, instead of concatenating the feature values to increase the dimension of the feature values. The results show that combining the extracted features from five different models was better than the concatenated features in predicting performance. The feature extract from the phylogenetic profile reflecting the biological significance of PHI in evolution was adopted. The results show that the feature values extracted by KPP can significantly improve the predictive performance of plant PHI. The KPP method extraction feature can be extended to predict the PHI of other organisms.

The performance of three plant PHI test datasets showed that the prokaryote organism of Psy (**Figure 3A** aucPRC = 0.685 for AA model) species performed better than the prokaryotes of Hpa (**Figure 3B** aucPRC = 0.574 for AA model) and Gor (**Figure 3C** aucPRC =0.618 for AA model) species in the test. It can be seen that the algorithm performed better for prokaryotes and less well for eukaryotes. About the human PHI test, the performance of human-bacteria PHI (aucPRC = 0.880) and human viruses (auPRC = 0.896) was better than the performance in the plant PHI test dataset (auPRC = 0.717 with AA model). auPRC of human bacteria is 0.880, and the auPRC of human viruses is 0.896. This also shows that the KPP algorithm that we developed can be applied to the prediction of PHI among different species and performs better for human PHI prediction.

We used the Gor-Ara, Psy-Ara, and Hpa-Ara training datasets for predicting the plant PHI. In the training dataset, the PPIN-1 proteins displayed high connectivity in AI-1MAIN and the PPIN-1 proteins as effector targets, in particular, are highly connected nodes within the overall plant network (Mukhtar et al., 2011). The protein TCP14 in plants interacted with 23 distinct Gor effector candidates, 25 Hpa effectors, and 4 Psy effectors that were the most targeted host protein (Wessling et al., 2014). Furthermore, TCP13, TCP15, and TCP19 were also targeted multiple times by effectors from at least two pathogens and exhibited altered infection phenotypes in the plant test dataset (Wessling et al., 2014). We identified SYNE1 (hsa:23345) and TTN (hsa:7273) genes as the hub genes in the host organism by predicting human-virus PHI (**Supplementary Table 7**). The SYNE1 genes encode a spectrin repeat-containing protein expressed in skeletal and smooth muscle, and peripheral blood lymphocytes; related pathways are meiosis and cell cycle, mitotic. The TTN gene encodes a large abundant protein of striated muscle. The diseases associated with TTN include myopathy and Salih myopathy. The SYNE1 mediates the docking of the capsid protein of human herpesviruses to nuclear pore complex proteins (Hong et al., 2021).

In the future, we hope that this approach will not only contribute as a useful predictor to accelerate the exploration of plant PHIs but also extend to the prediction of the PHI of more organisms.

## Methods

### KPP algorithm

#### Building the contig index and constructing the phylogenetic profile

Before creating a contig index, we needed to obtain a kmer set from *n* species proteomics. Here, the parameter *k*∈(1,2,3,…,*n*) and the kmer set are generated from the five different methods AA, HY, PO, CH, and CHP. A contig is composed of one or more consecutive kmers that are connected end to end. Building a contig index in advance can effectively compress the number of kmer and reduce the number of kmer backtracking queries, thereby improving the computational efficiency of feature extraction. We used the contig index to trace back whether the contig index existed in *n* species and generated a 0-1 (absence-presence) matrix as the binary phylogenetic profile.

#### Extract feature from phylogenetic profile

KPP cuts each pathogen and host sequence *S* to a kmer set and searches contigs *C*. For each *C* , we extract feature array  *A* from the binary PHI phylogenetic profile. The extracted feature function is defined as $f\left(C,A\right)=\sum_{i=1}^{C}A_{i}$ .

#### Combined method

Five model features extracted from the PHI phylogenetic profile have the same length. We propose a “combine” method to integrate the features for ML. The combined function is defined as $f\left(M,A\right)=\sum_{i=1}^{M}A_{i},$ where *M* is the feature extracted by the five different models. *A* is the feature array extracted from the PHI phylogenetic profile by various models.

#### Concatenate method

The feature extract from the phylogenetic profile concatenated with other methods to integrate features was defined as 〈*H*,*N*〉. Here, *H* is the feature array extracted from the phylogenetic profile. *N* is the feature extracted from other methods, for example, the structure-based method in this study.

### The structure-based method

#### DC method

DC represents the descriptor of two AAs in the protein sequence (Zhou et al., 2012). Dipeptide composition gives a 400-dimensional descriptor defined as $f\left(r,s\right)=\frac{N_{r,s}}{N-1}\;r,s=1,2,\ldots ,20,$ where *N*_*r*,*s*_ is the number of dipeptides represented by AA type *r* and type *s* .

#### CT method

The CT method is based on the percentage of three AAs in the sequence (Shen et al., 2007). Tripeptide composition gives a 343-dimensional descriptor defined as $f\left(r,s,t\right)=\frac{N_{r,s,t}}{N-2}\;r,s,t=1,2,\ldots ,7,$ where *N*_*r*,*s*,*t*_ is the number of tripeptides represented by AA type  *r* , *s* , and *t*.

#### AC method

The AC descriptor extracts features by accounting for the effects of the interaction of residues with a certain distance (Guo et al., 2008). The 210-dimensional calculation function was defined as $f\left(lag,j\right)=\frac{1}{N-lag}\sum_{i=1}^{N-lag}\left(X_{i,j}-\frac{1}{L}\sum_{i=1}^{N}X_{i,j}\right)\times \left(X_{\left(i+lag\right),j}-\frac{1}{N}\sum_{i=1}^{N}R_{i,j}\right)\;j=1,2,\ldots ,7,$ where *N* is the length of sequence  *X* , *j* denotes one descriptor, and *i* is the position in the sequence *X* . Here, *lag* ranges from 1 to 30 in this work.

#### PSP method

The PSP feature is based on protein secondary structure composition (Hoskins et al., 2006) and protein disorder information (Hsu et al., 2012; Meng et al., 2017) that was first proposed by Yang et al. (Yang et al., 2019). They calculated the fraction of three different secondary structure elements (a helix, b strand, and coil) and the percentage of disordered residues in three regions of the N terminus, C terminus, and the full sequence (Yang et al., 2019). Here, we calculate secondary structure and disorder information by PSSpred (Yan et al., 2013) and IUPred (Dosztanyi et al., 2005), respectively.

### Test data

The three different pathogens Gor (122 positive pairs), Hpa (104 positive pairs), and Psy (233 positive pairs) and also the negative pairs were downloaded from http://systbio.cau.edu.cn/intersppi/index.php (Yang et al., 2019). The criteria for choosing these three pathogens and Ara are that these interactions have been experimentally verified as real physical interactions. The experimentally verified human-bacteria (13,413 positive pairs) and human-virus interactions (14,789) were collected from HPIDB 3.0 database (Ammari et al., 2016). The positive interactions were filtered by “physical association” items in the PSI-MITAB(2.5) file while excluding the interactions betweenproteins with less than 30 AAs or nonstandard AAs. The sequences of the human bacterial and viral proteins were retrieved from the UniPort database (Consortium U 2014). Specifically, the ratio of negative pairs to positive pairs was 10:1. The proteomic data of species (503 species) for constructing the phylogenetic profile were downloaded from the KEGG database (Kanehisa and Goto, 2000).

### Performance evaluation

To conduct a stringent performance assessment, 10-fold cross-validation tests were carried out. We chose the precision-recall curve (PR curve) and the auPRC to assess the performance of our models. The formulas to calculate precision and recall are as follows:


$$Precision=PPV=\frac{TP}{TP+FP}$$

$$Recall=Sensitivity=TPR=\;\frac{TP}{TP+FN}.$$

## Data availability statement

The original contributions presented in the study are included in the article/**Supplementary Material**. Further inquiries can be directed to the corresponding authors.

## Author contributions

YY and CL conceived and designed research. YF implemented the software. YF performed the research. YY and CL drafted the manuscript and critically revised the manuscript. All authors read and approved the final manuscript.

## Funding

This work was supported by the National Natural Science Foundation of China (31870240).

## Conflict of interest

The authors declare that the research was conducted in the absence of any commercial or financial relationships that could be construed as a potential conflict of interest.

## Publisher's note

All claims expressed in this article are solely those of the authors and do not necessarily represent those of their affiliated organizations, or those of the publisher, the editors and the reviewers. Any product that may be evaluated in this article, or claim that may be made by its manufacturer, is not guaranteed or endorsed by the publisher.

## Supplementary material

Supplementary Figure 1 They effectively compress data by contigs. Compare contigs with kmer in five various models. The X-axis is the sequence of the protein sequence, and the Y-axis is the number of kmer or contig. **(A)** The effective compression of the AA model (k=8). **(B)** The effective compression of the HY model (k=22). **(C)** The effective compression of the PO model (k=27). **(D)** The effective compression of the CH model (k=19). **(E)** The effective compression of the CHP model (k=15).

Supplementary Figure 2 The predicted probability distribution of positive and negative pairs. **(A)** The predicted probability distribution of Gor-Ara. **(B)** The predicted probability distribution Psy-Ara. **(C)** The predicted probability distribution Hpa-Ara. **(D)** The predicted probability distribution of All-Ara. AA contig index model, the number of species was 503 and the kmer parameter setting was 6. The horizontal axis “Probability” is the output by random forest predicted result. The vertical axis “Percentage” is the proportion of the number of negative and positive sample pairs in the corresponding interval.

Supplementary Figure 3 The taxonomy of 503 species used to construct the phylogenetic profile.

Supplementary Figure 4 The performance of the combine and concatenate method. The auPRC of Gor-Ara, Psy-Ara, and Hpa-Ara by 10-fold-cross-validation are shown in **(A)**, **(B)**, **(C)**, and **(D)**, respectively.

Supplementary Figure 5 The performance of different ML methods. The auPRC of Gor-Ara, Psy-Ara, Hpa-Ara and All-Ara by 10-fold-cross-validation are shown in **(A)**, **(B)**, **(C)**, and **(D)**, respectively.

Supplementary Table 1 The classification of amino acids and five built phylogenetic profile models.

Supplementary Table 2 The influence of prediction performance by different species.

Supplementary Table 3 The list of 503 species for building the phylogenetic profile.

Supplementary Table 4 The influence of performance by different kmer lengths.

Supplementary Table 5 The performance of concatenating different phylogenetic profile feature models with the structure-based method.

Supplementary Table 6 The species of bacteria and virus and positive pairs.

Supplementary Table 7 Predictions of human-virus PHI pairs.

Supplementary Table 8 Predictions of human-bacteria PHI pairs.

## Abbreviations

PHI, pathogen-host interactions; ML, machine learning; KPP, kmer phylogenetic profile; Gor, Golovinomyces orontii; Hpa, Hyaloperonospora arabidopsidis; Psy, Pseudomonas syringae; Ara, Arabidopsis thaliana RF; Random Forest; AA, amino acid; auPRC, area under the precision-recall curve.
