# Supplementary figures and images for: New feature extraction from phylogenetic profiles improved the performance of pathogen-host interactions
Source: Front Cell Infect Microbiol. 2022 Aug 2;12:931072. doi: 10.3389/fcimb.2022.931072 (PMC9378789; doi:10.3389/fcimb.2022.931072)

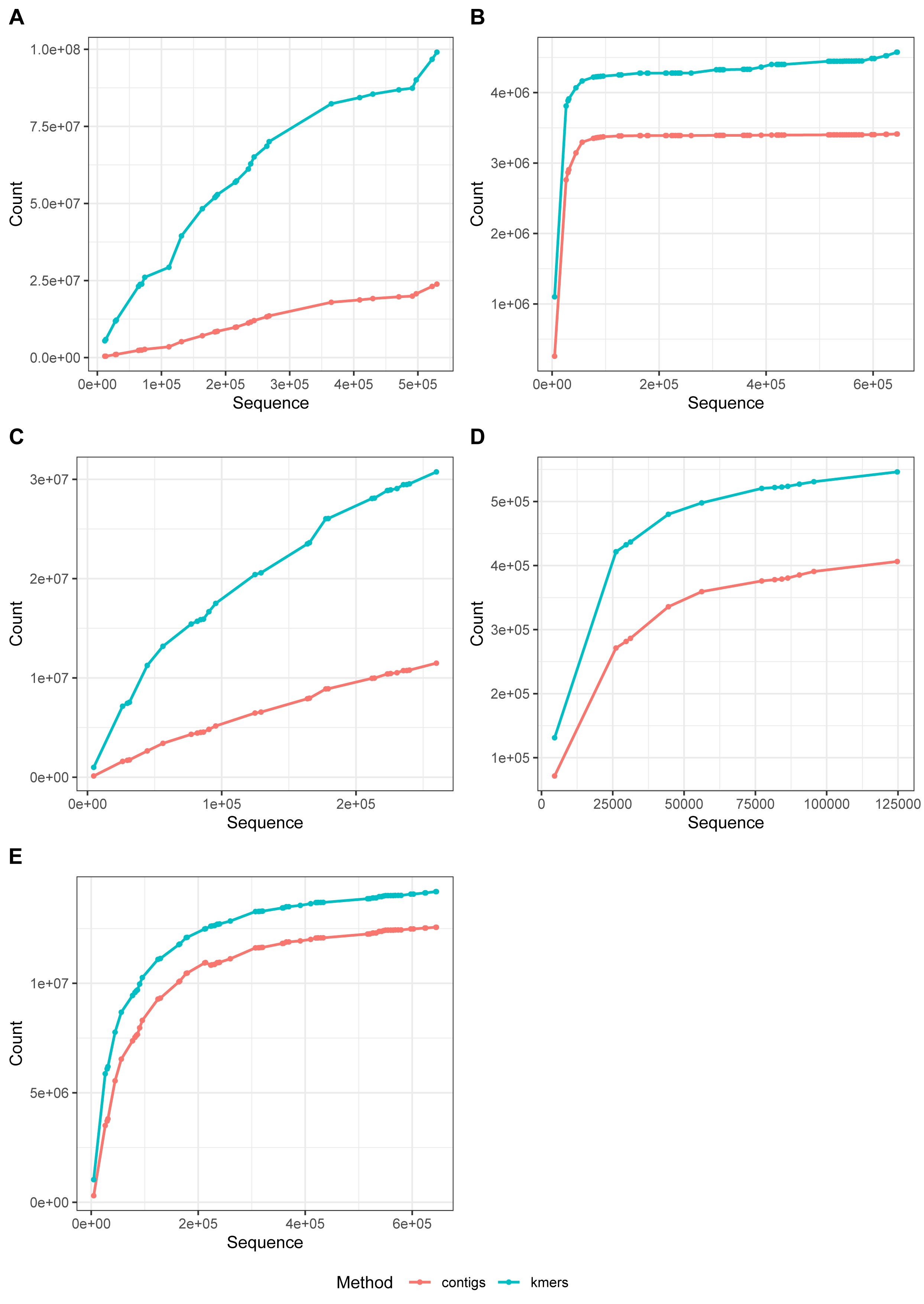

Supplement: Supplementary Figure 1 — They effectively compress data by contigs. Compare contigs with kmer in five various models. The X-axis is the sequence of the protein sequence, and the Y-axis is the number of kmer or contig. (A) The effective compression of the AA model (k=8). (B) The effective compression of the HY model (k=22). (C) The effective compression of the PO model (k=27). (D) The effective compression of the CH model (k=19). (E) The effective compression of the CHP model (k=15). [file Image_1.tif]

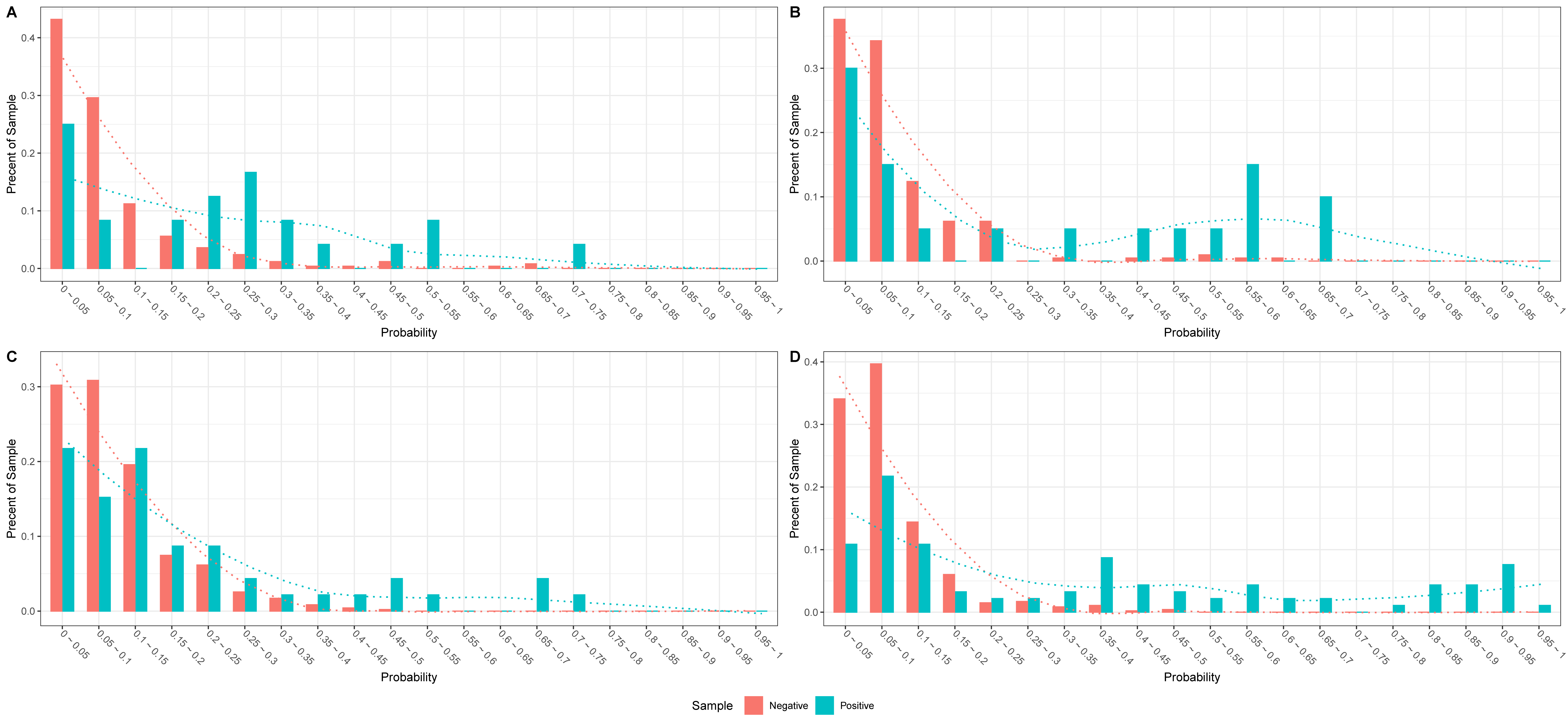

Supplement: Supplementary Figure 2 — The predicted probability distribution of positive and negative pairs. (A) The predicted probability distribution of Gor-Ara. (B) The predicted probability distribution Psy-Ara. (C) The predicted probability distribution Hpa-Ara. (D) The predicted probability distribution of All-Ara. AA contig index model, the number of species was 503 and the kmer parameter setting was 6. The horizontal axis “Probability” is the output by random forest predicted result. The vertical axis “Percentage” is the proportion of the number of negative and positive sample pairs in the corresponding interval. [file Image_2.tif]

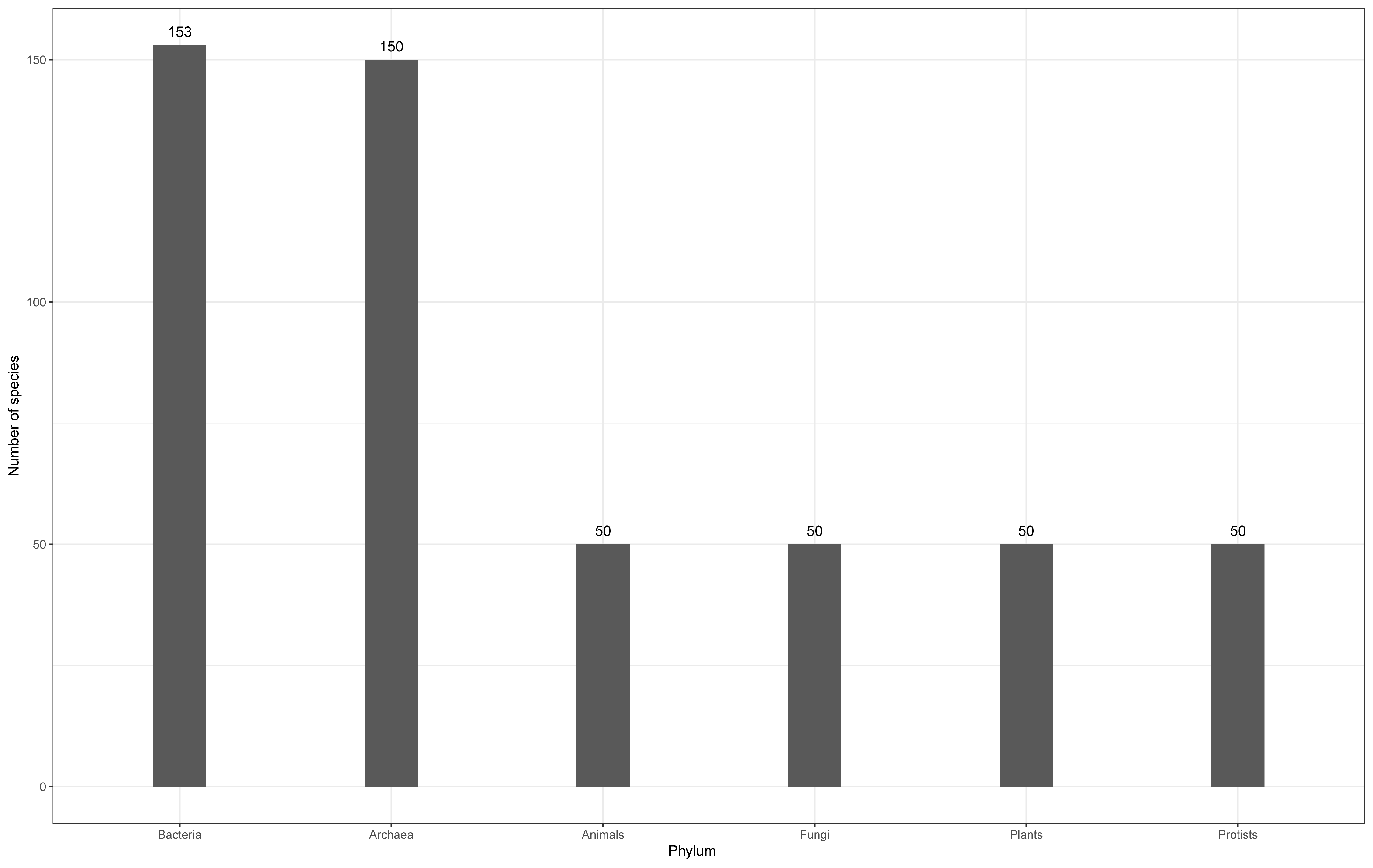

Supplement: Supplementary Figure 3 — The taxonomy of 503 species used to construct the phylogenetic profile. [file Image_3.tif]

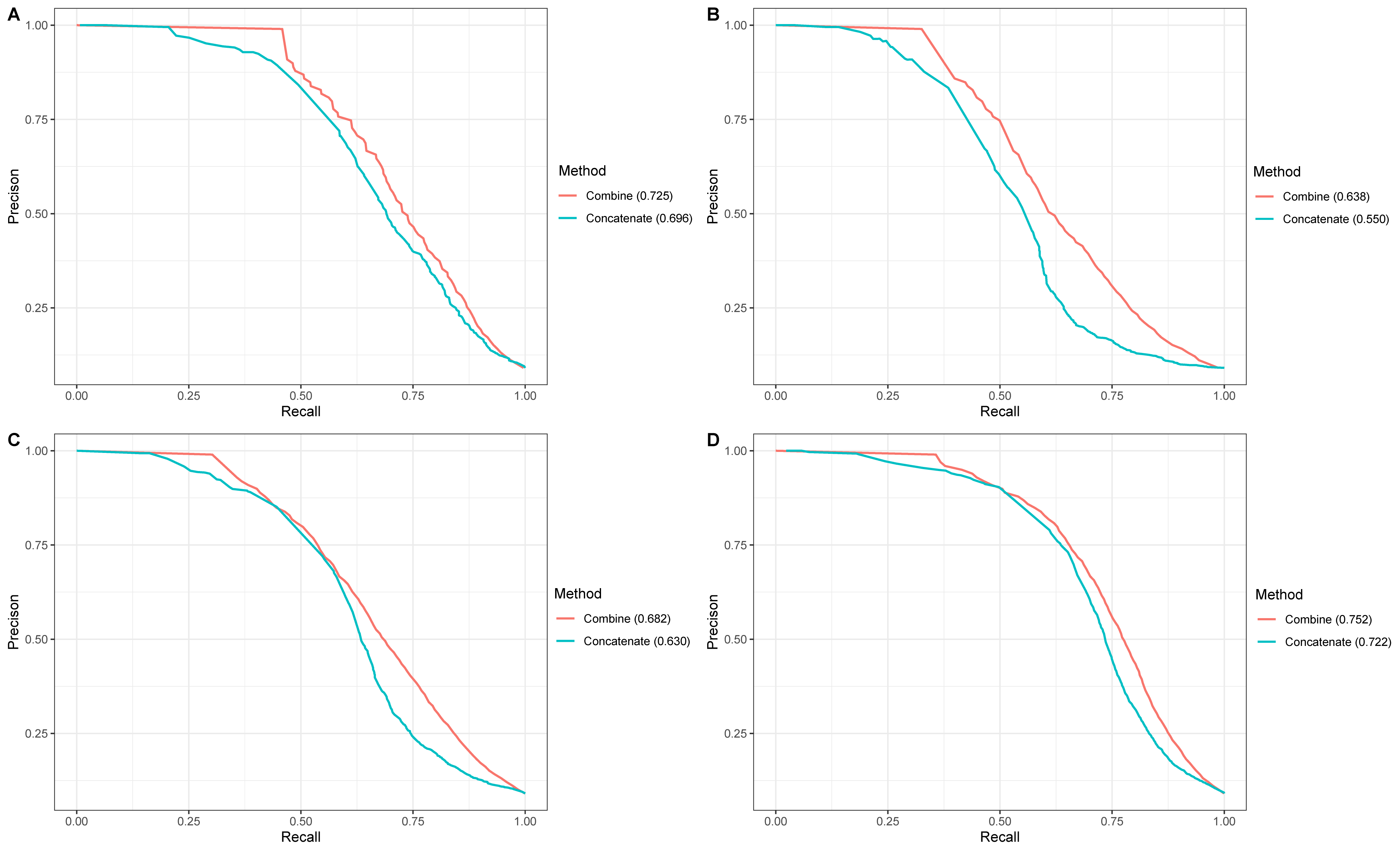

Supplement: Supplementary Figure 4 — The performance of the combine and concatenate method. The auPRC of Gor-Ara, Psy-Ara, and Hpa-Ara by 10-fold-cross-validation are shown in (A), (B), (C), and (D), respectively. [file Image_4.tif]

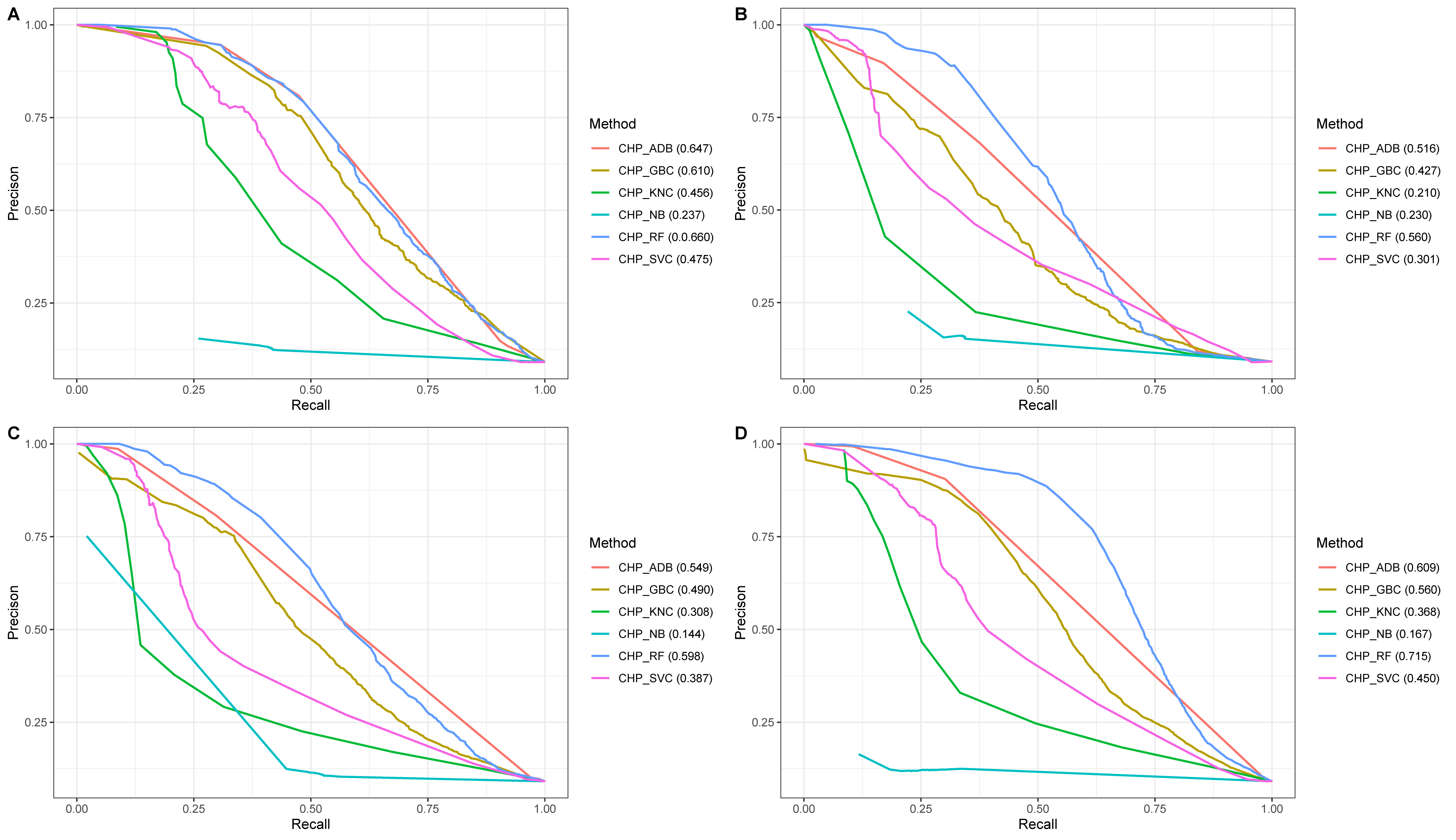

Supplement: Supplementary Figure 5 — The performance of different ML methods. The auPRC of Gor-Ara, Psy-Ara, Hpa-Ara and All-Ara by 10-fold-cross-validation are shown in (A), (B), (C), and (D), respectively. [file Image_5.tif]
